# The association of neighborhood deprivation with sleepiness among cognitively normal older adults with and without subjective cognitive decline

**DOI:** 10.1002/alz70857_107575

**Published:** 2025-12-25

**Authors:** Mark A Bernard, Arjun V. Masurkar

**Affiliations:** ^1^ NYU Grossman School of Medicine, New York, NY, USA; ^2^ NYU Alzheimer's Disease Research Center, New York, NY, USA; ^3^ Center for Cognitive Neurology, NYU Grossman School of Medicine, New York, NY, USA; ^4^ Alzheimer's Disease Research Center, New York University Langone Health, New York, NY, USA; ^5^ Center for Cognitive Neurology, New York University Langone Health, New York, NY, USA

## Abstract

**Background:**

Subjective cognitive decline (SCD) is considered a preclinical stage of Alzheimer's disease (AD). Sleep dysfunction, including excessive daytime sleepiness (EDS), is a known risk factor for Alzheimer's disease (AD) that may also increase SCD. We examined if neighborhood deprivation, a social determinant of health that may associate with poor sleep, increased EDS in cognitively normal older adults based on the presence of SCD.

**Method:**

This study included 81 cognitively normal older adults (mean age 73.9 years, 60.5% Female, 67.9% non‐Hispanic white, 43 with SCD, 38 without SCD) evaluated at the NYU Alzheimer's Disease Research Center. Participants underwent a comprehensive battery of psychometric tests according to National Alzheimer's Coordinating Center Uniform Data Set 3.0 standards. SCD was evaluated using the Global Deterioration Scale. EDS was assessed using the Epworth Sleepiness Scale (ESS). Neighborhood deprivation was assessed using the Area Deprivation Index (ADI). Multivariate linear regression models were used to evaluate the strength of associations between ESS score and neighborhood deprivation, with age, sex, education, and race as covariates. Models were run separately in those with and without SCD.

**Result:**

Among the study population at large, there was a statistically significant association between ADI and ESS scores (β = 0.490, 95% CI [0.138, 0.842], *p* =  0.007). This association remained when analyzing only participants without SCD (β = 0.609, 95% CI [0.181, 1.038], *p* =  0.007). Surprisingly, there was not a significant association between ADI and ESS in participants with SCD (β = 0.426, 95% CI [‐0.203, 1.055], *p* =  0.178).

**Conclusion:**

The impact of area deprivation on sleepiness depends on SCD status, with positive correlations only in those without SCD. This suggests a complex relationship between neighborhood disadvantage and other preclinical risk factors for AD. Further work should examine whether and how neighborhood disadvantage potentially impacts other aspects of sleep pathophysiology in persons with SCD.

